# A Practical Treatise on the Diseases of the Scalp

**Published:** 1842-10-01

**Authors:** 


A Practical Treatise on the Diseases of the Scalp. By *John E. Erichsen*, Member of the Royal College of Surgeons, &c. &c. Octavo, pp. 192, and Six Plates. London: Churchill, 1842.

Mr. Erichsen's object in bringing this Treatise before the public will appear from the following announcement in his Preface:—" I should feel that some apology were necessary in adding another to the already extended list of works on the Diseases of the Skin, were I not convinced that on the subject of the present treatise, the Affections of the Scalp, much error of diagnosis, and consequent confusion of treatment still exists. It is with the hope of remedying the one, and of simplifying the other, that this volume has been undertaken. It cannot, of course, be expected that much that is new can be advanced on a subject that has attracted the attention of so many eminent Dermatologists, and that has been so assiduously cultivated as this; but yet, I trust that by arranging what is already known on these diseases in a clear and methodical manner, by simplifying their diagnosis, and by showing how readily amenable they are, as much so as any other class of affections, to a rational mode of treatment, that the object I had proposed to myself might be accomplished.

" The writings of Willan and of Plumbe on the diseases of the scalp, excellent as they are in many respects, have appeared to me to tend to perpetuate the confusion that yet envelopes these affections, by their being grouped in them under the one term of ' porrigo,' a most artificial and unphilosophical nomenclature, by which the very purposes of classification are defeated; and a set of diseases differing from one another in nature, seat, character, causes and treatment, considered as species of the same genus, whilst, on the contrary, they belong to distinct orders. Why this practice of classing together all scalp affections into one group has been continued, it is difficult to understand; for although their distinctive characters are, to a certain degree, obscured and modified by local situation, by the presence of hairs, &c. yet if care be taken, they will be found to be sufficiently strongly marked not to be mistaken, by even a somewhat unpractised eye, and to enable the practitioner to refer them, without much difficulty, to the class to which they belong.

" Impressed with the conviction that these diseases will never be understood, until they are no longer considered as cases *per se*, but are placed in those orders to which their *elementary characters* point them out as belonging, I have, in the present treatise, discarded all *specific* names, and have arranged them in accordance with these characters. I have also endeavoured to direct the attention of the reader more particularly to their differential diagnosis, which is the only sure guide to a safe and rational treatment."—*Pref.* vii.—ix.

We fancy that, with a little transposition of words the identical complaints, hopes, regrets, and promises have been uttered by every writer on cutaneous disorders for the last twenty years. We shall see what Mr. Erichsen will do.

Over a Historical Introduction of thirty-four pages we may not inconveniently nor unprofitably pass. We then arrive at some "General Observations," from which we shall select what strike us. Prior to offering a classification of scalp diseases, Mr. Erichsen remarks:—

"Under the names of *scald head*, of *tinea*, or of *porrigo*, how many different diseases have there not been included? Diseases, such as impetigo, eczema, pityriasis, and favus, differing entirely in their nature, causes, effects, and treatment, and agreeing only in the part affected, have all been confounded together under one or other of these unfortunate terms. It certainly appears preposterous that a disease should go by a different name when it occurs on one region of the body than when it is situated in another."

However true this may be, there is certainly no absolute novelty in it, for we go no further than Rayer's book to find precisely the same views. We find in that, eczema, pityriasis, psoriasis, &c. described as they affect the scalp. This, then, the basis of the work, may be said to be a truism, inasmuch as all well-informed men have for some time past admitted and acted on it. It remains for us to see how Mr. Erichsen will develop the details of what is familiar in the general. His classification is this:—

"Scalp diseases arrange themselves naturally into the orders, *vesiculæ, pustulæ, tuberculæ*, and *squamæ*.

But two genera of the order vesiculæ, eczema and herpes, affect the scalp. The former of these is divisible into *acute* and *chronic eczema, eczema furfuracea* and *e. amiantacea*.

The genus *herpes* includes *h. circinnatus* and *h. zoster*.

*Impetigo* is the only pustular disease that ordinarily affects the scalp. When occurring in this region it is divisible into three species, viz. *impetigo eczematosa*, the impetigo or porrigo larvalis of others; *impetigo granulata*, the porrigo or tinea granulata; and *impetigo sparsa*, the porrigo favosa of Willan.

For reasons to be afterwards explained, I have thought that *favus* could with more propriety be considered a tubercular than a pustular affection, meaning by *tubercular* not a disease, like lupus, characterized by the presence of small tumors, but one in which true tubercle is deposited; I have therefore removed it from the order pustulæ, and have placed it by itself.

The only squamous disease that commonly occurs on the scalp is pityriasis. Lepra and psoriasis are also occasionally, though rarely, seen in this region."

Of course on the scalp, as on the other parts of the body, these orders of cutaneous alteration run into one another.

Mr. Erichsen states, we think with truth, that the local condition in scalp affections is, for the most part, inflammatory. This, however, does not seem to be the case with Favus.

Speaking of the local and constitutional causes of cutaneous diseases, Mr. Erichsen seems to us to speak too doubtingly of their connexion with disorders of the digestive organ. "There has certainly," he says, "been too great a stress laid by some writers upon what has been considered to be a sympathetic connexion between the digestive organs, more particularly the liver, and the skin. But although we may question whether this connexion exists to the extent that has been supposed, still it would be unwise to reject it altogether, as we continually meet with instances in which one of the first indications of a derangement of these organs is an eruption of acne, of herpes, or of impetigo, especially about the face, which has been immediately removed on a restoration of the normal action of these viscera." We think it is impossible for any practical and experienced man to doubt the connexion, more frequent and more intimate than Mr. Erichsen is willing to allow, between disorders of the digestive organs and affections of the skin, as well as between some morbid condition of the humours and the latter.

Our author admits that scalp diseases are not so contagious as has been imagined. But of the ready transmission of favus by contact there can be no question; for notwithstanding the opinion of Alibert, that English physicians had been too hasty in admitting this, it is now most satisfactorily proved by numberless cases. Eczema seems also, in some instances and under peculiar circumstances, to be contagious.*

"Individuals of a scrofulous diathesis are peculiarly liable to most of the diseases of the scalp, but more especially to favus, simple chronic eczema, impetigo eczematosa, and impetigo sparsa. By far the majority of children labouring under these affections, will be found to present what are usually considered to be marks of a scrofulous habit of body, at least the fair variety of it; that form of it characterised by a dark sallow complexion, predisposing them rather to impetigo granulata. As diathesis is so intimately connected with the hereditary transmission of disease, we should expect that in many cases there would be an hereditary predisposition to affections of the scalp, parents transmitting to their offspring the diathesis, and consequently the disposition that it gives to particular classes of disease. It is said that favus has been, in some rare instances, found to be congenital, but this is doubtful, and requires confirmation.

The frequency of the occurrence of the diseases of the scalp, and the kind of disease that is likely to occur, their absolute and relative frequency, is much influenced by age; the earlier years, especially the period of the first and second dentitions, being by far more subject to these affections than any subsequent age, although they are occasionally met with after puberty, and even in advanced life. The nature also of the affection that will probably manifest itself is much influenced by the age of the individual, thus impetigo eczematosa is most frequently met with before the third year, whilst impetigo granulata is rarely seen until after that period. At the early age at which scalp diseases usually occur, sex appears to influence the constitution but little, and consequently does not, as in after life, predispose to any particular affection.

Eczema amiantacea seems to be the only one of these diseases that is under the influence of moral causes. How these, more particularly the depressing passions, act in giving rise to this affection is uncertain, but that they do so, is established beyond a doubt by the concurrent testimony of all those who have had an opportunity of studying this complaint." 15.

Mr. Erichsen makes some general remarks on the *Treatment* of Scalp Diseases. He observes, that the local treatment presents four indications.

---

* "Pujol relates a remarkable instance of this. A dentist labouring under acute eczema of the hands, infected in one day the faces of a number of pupils at the Military Academy of Sorèze.—(Rayer.)"

"1st. To prepare the scalp for the application of topical remedies by the removal of the hair and scabs.

2nd. To lessen any irritation, or inflammatory excitement that may exist.

3rd. To excite the part to a new action by the employment of proper local means.

4th. To continue the use of the remedies for some time after the disease has been, to all appearance, cured."

1. The removal of the hair is *the* first thing. If the head can be shaved so much the better. If not, the hair should be cut close.

"In some affections, as in favus, the hairs act as foreign bodies in the diseased follicles, and it therefore becomes necessary to remove them from their attachment to the bulbs. This used to be effected by means of that barbarous application the pitch cap, and more recently by the equally cruel one, of pincers; but both these methods may be entirely superseded by the use of gentle depilatories, which effect the same purpose without pain or trouble. The most useful of these are alkaline ointments and lotions, which if applied every evening, or morning and evening, will in the course of a very short time cause the hairs to be sufficiently loosened, to be removed by means of a small-toothed comb passed repeatedly and lightly through them, after they have been cut to the length of an inch or an inch and a half." 20.

To get rid of the scabs is most easily effected by the "application of a large bread and water poultice which should be covered with a piece of muslin or of gauze, so as to prevent the crumbs from adhering to any hairs that may have been left. Linseed-meal poultices are objectionable, as they sometimes set up a considerable degree of irritation, giving rise even, in some tender scalps, to an eczematous eruption. Fomentations may be employed for the same purpose, but unless much inflammation be present they are not so serviceable as poultices, with the use of which, however, they may frequently be alternated with advantage." Ointments are apt to become rancid and irritating. But we have seen them of great service in subduing irritation notwithstanding. We agree with Mr. Erichsen, "that the use of poultices and fomentations, one or both, should be persevered in until all the inflammation that accompanies the onset of eczematous and impetiginous affections of the scalp has been subdued, and as long as this state continues no other local means should be resorted to; stimulating applications of all kinds being especially avoided, as they cannot possibly be of any service, and are constantly productive of the most mischievous effects; keeping up for weeks, and months even, a disease that, under rational treatment, would subside in the course of a few days."

2. Irritation and inflammation are relieved by the same means that promote the detachment of the scabs. Depletion, local or general, *may* be necessary.

3. The next object is to set up a new action in the vessels of the diseased scalp. On this point, Mr. Erichsen observes:—

"The alkalies and their carbonates, more especially the preparations of potass, exert a very beneficial influence on many of the more chronic diseases to which the scalp is liable. It will be found, that during their use in the chronic forms of eczema and of pityriasis, the scalp will very quickly be freed from the scurf and scales, which in these affections cover it to a considerable extent, and that the skin will very soon assume a clean, smooth, healthy appearance, these preparations appearing both to act chemically upon the epidermis, and to stimulate the cutis to a more healthy action. In the form of ointment they are very useful in the treatment of favus.

The alkaline sulphurets are equally serviceable, and in the treatment of scalp diseases supply the place of sulphureous baths. The sulphuret of potassium, in the proportion of one or two drachms to a pint of water, will be found more generally useful than any other local application in those chronic cases of eczema or of impetigo in which there is but little inflammatory action going on, and which are attended by no discharge. As these preparations are powerful stimulants, exciting the vessels of the skin to an increased activity, their use is contra-indicated as long as any active inflammation exists, as it would infallibly be augmented by them.

Lotions containing the metallic salts, such as the sulphates of copper or of zinc, or the nitrate of silver, may be employed with marked benefit in those cases of eczema which are attended by a considerable oozing of serous fluid from a multitude of small openings, without the formation of any fresh vesicles. In some very chronic and indolent cases of this description, the strength of the solution of the nitrate of silver may be increased to ten or fifteen grains, or even to a scruple of the salt to an ounce of water, with marked advantage. A lotion of this strength sets up an active inflammation in the part to which it is applied, that appears to destroy or to modify favorably, the chronic action that is going on, for when the artificial inflammation is subdued, the scalp will in general be found to revert to a healthy condition without any recurrence of the disease. The inflammation being however, for the time, greatly increased, some alarm is apt to be excited in the mind of the patient, who imagines that the means adopted for his cure are only augmenting the severity of his disease; it therefore becomes necessary to put him upon his guard in this respect. When these lotions are employed of a milder strength they appear to arrest the discharge by constringing the vessels of the scalp, and thereby removing that passive, atonic state which is so common a consequence of chronic inflammations, especially in scrofulous lymphatic subjects.

Various ointments are employed in the treatment of these diseases; they should however be dispensed with as much as possible, as the application of a greasy substance, which is frequently rancid, very commonly increases any inflammation that may already exist. I have certainly seen more harm than good result from the use of these preparations in eczema and impetigo of the scalp, in which diseases lotions may be substituted with advantage, as they are less likely to irritate the skin, besides having the additional recommendation of being much more cleanly and agreeable to the patient. In cutaneous affections, ointments should only be employed for the purpose of stimulation; a soothing ointment is a misnomer, for as the grease, which necessarily enters into its composition, is always an excitant to the skin, no substance with which it can be combined can exert a soothing or sedative influence upon that tissue, whatever it may upon the system generally. Of the ointments of the Pharmacopœia, those of the nitrate of mercury, of the nitric oxide, and of the ammonio-chloride of the same metal, will be found to be the most useful, and, according to the degree of stimulus required, to answer every purpose. As has already been stated, the carbonate of potass, when made into an ointment, is an excellent depilatory. The preparations of iodine, such as the iodides of sulphur, mercury, lead, and arsenic, may also be employed in a similar form with marked benefit. The iodide of sulphur, in the proportion of from twelve grains to half a drachm, to an ounce of lard, exerts a most beneficial and energetic action in chronic cases of favus, more particularly when it is combined with the occasional use of alkaline lotions. It is to Biett that we owe the introduction of this preparation in the treatment of the diseases of the skin, and it certainly has proved to be a most useful auxiliary to the means we already possessed." 26.

Wé observe that Mr. Erichsen repeats the cuckoo-note about *grease.* This appears to be a dogma at University College, and Mr. E. is, we suppose, bound "jerare in verba magistri." The absurdity of denouncing "soothing ointments" will be palpable enough to whoever has laboured under affections of the skin attended with much itching or irritation. The effect of lotions in relieving either is generally very inferior, and the ointment of zinc and lead in nine cases out of ten soothes more than all the washes that ever were composed.

The *General Treatment,* according to our author, comprises four indications:—

"1st. Lessen or subdue any excitement or inflammatory action that may be going on.

2d. Modify or remove the morbid action set up in the economy, on which the local disease is dependent.

3d. Lessen irritation.

4th. Support the powers of the system when they have suffered in consequence of the profuseness of the discharge, or of the irritation produced by the disease." 27.

Purgatives are, of course, insisted on; the aqueous extract of aloes, combined with a mercurial, and followed by a saline aperient, is recommended.

Among the alteratives the preparations of iodine are lauded. Mr. Erichsen writes:—"The iodide of iron, in doses of a quarter of a grain to two grains three times a day, will be found to be of essential service in cachectic or scrofulous habits, appearing to conjoin the advantages of a tonic with those of an alterative. When given in combination with those bitter infusions with which it is compatible, it is a most valuable adjunct means in the treatment of those cases of favus and of eczema that occur in scrofulous subjects. Its administration requires, however, some little care, as it is apt to excite the system."

Mr. Erichsen speaks favourably of the sulphuret of potassium. It forms, he says, a very active preparation, which may be employed with advantage, instead of the Harrogate or other sulphureous waters, in doses of from five to fifteen grains in a glass of water. The bowels should at the same time be kept gently open with small doses of some saline aperient, such as the tartrate of potass or the sulphate of magnesia, as otherwise the sulphuret of potassium is apt to stimulate the system too powerfully. The chief objections to its internal use are its nauseous taste and odour, which are usually very potent objections indeed.

To allay irritation, Mr. Erichsen recommends the tincture of hyoscyamus and dilated hydrocyanic acid. He adds:—"The mineral acids are also of great use in allaying the itching and tingling that so commonly occur in the impetiginous and eczematous affections of the scalp. The diluted nitric acid may be given with this view in doses of from ten minims to half a drachm, and the diluted hydrochloric acid in doses of from twenty minims to a drachm, in a glass of barley-water, or with a little syrup of capillaire, or of orange-peel, twice a day. After they have been continued for some time, their employment must be intermitted for a few days, as otherwise they are very apt to give rise to some irritation of the digestive organs."

We confess that we have seen more harm than good from the mineral acids in eczema. They have in many instances aggravated the irritation and produced an exacerbation of the eruption.

When the powers of the system have been reduced, tonics are necessary.

Throughout his observations Mr. Erichsen makes no mention of the liquor potassæ. Yet we venture to say, that, judiciously and boldly exhibited, it is worth all the physic he has spoken of put together. And while he enumerates the mineral acids and quina and iron, stimulating tonics that are as likely to do harm as good, he makes no allusion to the mild and much more safe and valuable tonic sarsaparilla.

### Vesicular Diseases.

These form the subjects of the second chapter.

Mr. Erichsen follows Biett in dividing eczematous affections into *acute* and *chronic,* the latter comprising *simple chronic eczema, eczema furfuracea,* and *eczema amiantacea.* This division is certainly the most simple and practical, as the chief indications in the treatment of this disease vary according as it is acute and active, or chronic and passive, in its nature.

*Acute Eczema of the Scalp.*—Mr. Erichsen gives an accurate description of this. "The appearance," he says, "of any eruption in this disease is always preceded by a sensation of heat and tension in the scalp with some itching and tingling; if the part be now examined, a red blush will be observed upon it, and its temperature will be found to be sensibly augmented. This exanthematous blush, which always precedes acute eczema, frequently escapes attention in the first attack of the disease, but it may be observed to be of constant occurrence in those cases that supervene upon a chronic form of it. After it has continued for a few hours, an eruption of small vesicles makes its appearance, and the nature of the affection is at once recognizable. These vesicles, which are at first very small, gradually enlarge until they attain the size of a pin's head. On their appearance the pruritus and tingling is usually somewhat lessened, but this is not always the case. They are for the most part evolved in patches, each of which contains a number of them very closely set together, but sometimes they occur in large clusters. The fluid contained in them is at first clear, limpid, and either colourless or else of a slightly yellow tint; it soon, however, becomes turbid and milky in appearance, and if the vesicle remain two or three days without breaking, or being ruptured by the patient scratching the part, it will be found to have assumed a puriform aspect.

The vesicles are at first always somewhat pointed in figure, but as they become larger they may assume a spherical or globular form; they are situated between the hairs, which consequently do not traverse them, although this appears occasionally to be the case when two or three of them have run together inclosing a hair, as it were, in their centre. Sometimes the fluid contained in them appears to be re-absorbed, leaving merely thin, white, scaly incrustations on the surface of the scalp, which, from their close resemblance to, have often been mistaken for the scales of pityriasis. More commonly, however, on the second or third day, the vesicles give way, and their contents being effused, form, on drying, small, rather thin, yellowish-white or grayish scabs, which mat and bind the hairs together. If the disease be not arrested in this early stage, a thin semi-transparent fluid having some resemblance to lymph will continue to exude, frequently without the formation of any new vesicles, from numberless small openings left in the site of the old ones. This fluid, if in large quantity and not very viscid, soaks the hair and forms it, especially about the roots, into small bundles, which are soft, moist, and of a dirty yellowish-grey colour. If the quantity of secretion be not quite so great as this, it dries into scabs, which, by being constantly added to the former ones, gradually cause them to acquire considerable thickness and size. The growth of the hairs, that are entangled in these scabs, gradually separates them from the scalp. This process is rather curious, the scabs being always lifted off in the direction of the growth of the hair; thus those that are situated on the fore-part of the head are first raised off at their anterior part, whilst those, on the contrary, on the occipital region are first of all loosened at their lower aspect. When the scab is completely separated from the skin, it becomes dry, assuming a white pulverulent appearance, gradually crumbling and breaking away."

With respect to the treatment of acute eczema we see nothing to detain us.

*Chronic eczema* is divisible into three species :—

" 1st. *Simple chronic eczema,* which may be either moist or dry.

2d. *Eczema furfuracea,* corresponding to the *porrigo furfurosa* of Willan, the *tinea furfuracea* of others, and the *achor lactuminosus* of Alibert.

3d. *Eczema amiantacea.* The *teigne amiantacée* or *porrigo asbestina* of Alibert, who was the first to describe this form of the disease.

These are not necessarily preceded by an acute attack.

*Moist Chronic Eczema* is thus described by our author :—" There is always a very copious discharge of a thin serous fluid from a number of small openings in the scalp, which are usually very closely set together and very numerous. This discharge, which is frequently of an acrid and irritating nature, is apt to increase the inflammation in that part of the skin on which it is allowed to remain, or over which it flows. If it be very abundant, the hair looks as if it had been soaked in a thin solution of gum arabic, being matted together in locks, which have a dirty yellowish-gray moist appearance, and between and under which, the inflamed scalp may be seen to be perforated by a number of minute openings, which pour fourth the discharge. As it lessens in quantity soft yellowish-gray scabs will be formed, which gradually losing their moist appearance, will be found to resemble those that characterize the dry variety of the disease. In the midst of this, acute attacks of eczema, attended by a fresh evolution of vesicles, by increased heat and redness of the scalp, frequently occur, adding very greatly to the severity and obstinacy of the disease. A peculiar pungent acid odour, somewhat resembling that of the fumes of acetic acid, is at the same time evolved from the head, and the distress of the patient is often greatly increased by a chronic inflammation of the eyes and ears, which is very apt to occur in this form of eczema. As the discharge lessens, the moist, in many cases, gradually passes into the dry variety of the disease."

2. *Dry Chronic Eczema* may be very limited or extensive on the scalp. "The disease," says Mr. Erichsen, "is characterized by scales or scabs of a yellowish-white, yellowish-gray, or yellowish-green colour, which are darker in their centre than at the circumference; they are usually of an irregular figure, but some of them frequently assume a rhomboidal or diamond shape, being separated from one another by cracks and fissures, at the bottom of which may be seen the inflamed scalp, covered by a mealy powder, the detritus of the scabs; they are always more or less lamellated, and are usually about a line or two in thickness, being thinner at the circumference than at the centre, which is the part that is last of all separated from the scalp. They are loosened from the skin by being gradually pushed up by the growth of the hairs, the part that is farthest from the vertex being that which is first of all raised up, as has already been stated; the sides are then separated, and when the upper and middle parts are detached, the scab falls off, leaving the subjacent skin dry, glazed, and of a bright red colour. They occasionally present a depressed appearance in the centre, from their sides being curled up, but this is very different from the cupped shape of the crusts of favus. They are usually most numerous about the vertex, on the sides of the head, and about the ears. The hair, which is commonly dry, thin, and brittle, readily breaks off, but as it returns to its original strength and beauty when the disease is cured, no permanent baldness is left."

The two forms, however, run into one another, more especially the moist into the dry.

Chronic eczema may last for a very long time, even for several years.

In the treatment of chronic eczema, after the scabs have been detached by poultices, local stimulants are necessary. For this purpose, observes Mr. Erichsen, we may either employ the alkaline carbonates or sulphurets, or lotions and ointments containing metallic preparations; the former sufficing in the milder cases, the latter being required when the disease has become more obstinate, in consequence of neglect or of injudicious treatment.

Lotions, containing either the pure alkalies, or their carbonates and sulphurets, will be found to be especially useful in cases of dry chronic eczema, after the scabs have been removed by poulticing, and the surface of the scalp been thoroughly cleansed. The quantity of the liquor potassæ, carbonate of potassa, or sulphuret of potassium, used, should vary from one to three drachms to a pint of water. If any inflammation be excited during the employment of these preparations, they must be discontinued until this be subdued.

If, however, the disease prove to be more rebellious, and especially if it be the moist chronic form of eczema, we must either substitute for, or alternate with the employment of these means, an ointment made with some of the metallic preparations, such as the nitrate of silver, the iodide of sulphur, the bichloride, nitrate, or ammonio-chloride of mercury, or the sulphate of zinc, according to the degree of stimulation that the scalp will bear. The mode of employing these, that Mr. Erichsen has found to be most beneficial, is to apply the ointment, that we determine upon using, at night, ordering the patient to wash it off in the morning with a lotion composed of the sulphuret of potassium, then to re-apply the ointment, and in the course of six or eight hours to wash it off again ; thus alternately re-applying and washing off the ointment at stated intervals during the four-and-twenty hours. This alternation of stimulus will prove to be exceedingly useful ; but it will in general be found that, any one local application which, at first appears to be of great service, will, after a time, lose its effect and necessitate the employment of some other.

Our author objects to an oiled-skin cap. He prefers one of thin linen.

The *general treatment* must be regulated by circumstances, and in correspondence with the symptoms.

*Eczema Furfuracea.*—"This disease presents considerable variety of appearance, according to its extent and intensity. When slight and of but trivial extent, it appears in the form of a few thin scales of a yellowish-white or yellowish gray colour, which are the remains of vesicles that have either shrunk without effusing their contents, or else have contained such a minute quantity of thin serum, that when it has dried up it has been insufficient to form a scab. These scales are sometimes placed upon the summits of small papillæ, the tops of which occasionally present a black appearance, in consequence of the drying of a minute quantity of blood that has been effused by the patient scratching himself. When the disease assumes this slight form, it would scarcely merit the attention of a medical man were it not frequently the forerunner of a more serious attack.

The more severe forms of the disease are usually preceded by a considerable degree of pruritus, with some tension and heat about the scalp; the epidermis then appears to separate itself into detached scales, which are often loosened by the patient scratching himself; after which a few small vesicles arise, from which a thin, serous, but rather glutinous fluid exudes, forming the scales into masses, and agglutinating them and the hairs together. These masses of scales and hair give a soft, yielding sensation to the finger when they are pressed upon, and if they be removed, we shall find the cutis underneath to be exposed and inflamed.

These scales vary in colour from nearly a pure white to an ash-gray, yellowish-gray, yellowish-brown, and brown, presenting, when of the latter hue, a very close resemblance to bran. The depth of colour depends more upon the quantity of fluid with which they are impregnated than on any other cause, as the same scales which are dark when moist, become light-coloured when dry.

This disease may be confined to patches on the scalp, or it may extend over the whole of the head, and even down upon the forehead; when extensive and of old-standing, the head evolves a disagreeable cheese-like odour. The hairs are never lost, but occasionally become thinner and lighter in colour. As the disease declines, the discharge dries up, and the scales fall off, separating in large quantities when the patient shakes or scratches his head, so that it is impossible for him to keep his clothes free from them, the pruritus ceases, the scalp gradually assuming its natural appearance." 63.

*Eczema Amiantacea.*—"This disease, which is a frequent consequence of the preceding one, is characterized by a mass of white, pearly scales, of different sizes, which adhere to and surround the roots of the hairs, matting them into locks, and giving to the head a white, striated glistening appearance, which makes it resemble sufficiently closely dirty coarse amianthus or asbestus.

The symptoms of this disease vary according as to whether it comes on after an attack of the preceding affection, or whether it occurs in an acute form, presenting from the very onset its asbestiform appearance.

In the former case, the scales of eczema furfuracea gradually become more numerous, and are collected into thicker masses, which by drying on the surface present that peculiar gray, pearly appearance which is so characteristic of the disease, the deeper layers, however, retaining their soft, glutinous feel. In the mass which is thus formed by an agglomeration of hairs, of serous discharge, and of scales, the individual hairs cannot be discerned, as they are collected into bundles, which may again be sub-divided into smaller ones, but which it is impossible to resolve into the separate hairs that compose them. When these locks are cut off, and the scales removed by poulticing, the scalp will be seen to be of a vivid red, and to pour out the serous discharge that binds the hairs together." 64.

We confess that we see little advantage, but rather the reverse, in separating eczema furfuracea and eczema amiantacea. It is this disposition to give names and individual descriptions to affections that constantly run into one another, and differ only in some trivial point, that has contributed, we think, to keep back the amount of useful general information on the subject of cutaneous complaints.

These diseases may last weeks or years. "When eczema furfuracea commences in infancy, it usually disappears about the period of puberty, but it may continue after that time of life; when this is the case it assumes a very obstinate character. Eczema amiantacea is always a very chronic complaint, and one that is with difficulty influenced by any mode of treatment."

Stimulating lotions of various sorts have been employed, with indifferent success, in the treatment of these disorders. Mr. E. lauds the solution of the nitrate of silver, in the proportion of a scruple or half a drachm to the ounce of water, pencilled over the scalp every second day.

*Herpes.*—After a description of herpes circinnatus, the "ringworm" of the public, an affection neither contagious, nor communicable by inoculation, Mr. Erichsen touches on the necessity of its diagnosis, and points out the features of it. The disease may be distinguished from all other affections of the scalp by the circular or oval figure that its patches assume, and by the vesicles being placed exclusively on the circumference of the ring, leaving the centre free. These characters will prevent its being confounded with *eczema,* which disease never appears in patches of an oval or circular form.

From *favus,* the true ringworm of the scalp, there can be no difficulty in distinguishing this disease at a glance. For although they both assume a circular form, yet herpes circinnatus is a vesicular disease, with thin small scales, and without any tendency to occasion a permanent loss of hair; whilst favus, on the other hand, is a tubercular affection, with yellowish-gray crusts, and is usually attended or followed by a permanent destruction of the hair-bulbs, being altogether a much more severe disease. Its vesicular characters are sufficient to prevent its being mistaken for any of the *impetiginous* affections of the scalp. *Lepra,* in its decline, might at first be confounded with it, but this is a squamous disease, and moreover, seldom, if ever, occurs in single patches, so that any doubt that might arise from the appearance of one circle, might easily be cleared up by seeing others in different stages on other parts of the body.

Speaking of herpes zoster, and its abrupt termination at the median line, Mr. Erichsen offers an hypothesis to account for that curious circumstance.

"The occurrence of herpes zoster on one side of the body only, and its abrupt termination at the median line, can, I think, only be explained on the supposition that the nerves of the region affected exert a direct influence upon the disease. In support of this opinion it will be found that herpes zoster always follows the course of the nerves of the part on which it occurs; thus, when it is seated on the trunk it almost invariably curves downwards and forwards in the very direction of the intercostal nerves, and several cases have fallen under my observation in which it has occurred in the precise course of the sciatic nerve. This view of the nature of the disease is strengthened, when we take into consideration the severe neuralgic pain that is so commonly left on its disappearance, and which clearly indicates some local morbid condition of the functions of the nerves. Its abrupt cessation at the median line also resembles what takes place in some nervous diseases, a hermicrania for instance. On taking all these circumstances into consideration, it is, I think, impossible not to come to the conclusion that herpes zoster is essentially connected with some local derangement of the nerves of the part affected." 77.

### PUSTULAR DISEASES OF THE SCALP.

Under this head Mr. Erichsen ranks *impetigo* only. Favus he places under a different order.

*Impetigo.*—This is extremely common on the scalp. It comprises three species, viz. impetigo sparsa (the *porrigo favosa* of Willan;) impetigo granulata (the *tinea* or *porrigo granulata*); and impetigo eczematosa (the *porrigo* or *impetigo larvalis*): the first being characterized by *psydracious*, the last two by *achorous* pustules, and differing besides from one another in some very important respects.

*Impetigo Sparsa.*—Mr. Erichsen gives a good account of the symptoms of this affection. "The appearance," he says, "of pustules in this disease is preceded by an erythematous blush and by a considerable degree of tingling and itching, which is sometimes very intense and distressing to the patient. After the lapse of a few hours a number of elevated points appear on the inflamed skin; these, which are at first very smooth and shining, soon become converted into pustules, which may be either scattered singly upon the scalp or distributed here and there in small groups. These pustules vary considerably in size from that of millet-seed to a split-pea, partaking in the latter case of the characters of phlyzacia; but in general they are small and appear seated in the structure of the cutis, and not upon it; they are usually most numerous about the posterior part of the head and vertex. The itching and tingling are but little if at all diminished on their evolution, in fact, in some cases, they appear to be increased, and the erythematous blush that preceded them continues of the same intensity as before.

In from forty-eight to sixty hours after their formation, these pustules give way and shed their contents upon the surface of the scalp, where, concreting rapidly, they form thick irregular scabs, of a yellowish-green or yellowish-brown colour, semi-transparent, and having a varnished glistening surface, which causes them to resemble very closely masses of impure gum-arabic or of dried honey. As the oozing of pus continues, these scabs increase in thickness and gradually acquire a rounded, mamillated, or stalactitical form. They are adherent to the scalp, and are usually traversed by several hairs that have been entangled in them. After a time they crack, becoming dry and friable at the edges where they assume a light yellowish-gray colour, and an opaque appearance, and gradually crumble away. By the continuance of the discharge and the rapid concretion of the effused fluid, large masses of them soon form, which, uniting together, give rise to continuous incrustations, around which, pustules may frequently be observed in all the stages of their progress."

Serum or even pus may be poured out in the subcutaneous cellular tissue, and the lymphatic glands often swell, but seldom suppurate. When the complaint is on the decline, the formation of pustules ceases, the scabs already formed separate in an irregular manner, and are either not reproduced at all, or if so, not to the same extent as before. The surface, that is exposed by their falling off, will be seen to be red and shining, deprived more or less of hair, and covered by a thin glistening cuticle, which soon, however, becomes thicker and stronger. Any baldness that may be left by this disease is only temporary.

*Impetigo Granulata* commences " by the eruption of a number of small isolated, achorous pustules, of a light yellow colour, which are usually scattered irregularly upon the surface of the scalp, and not congregated in clusters; each pustule being traversed by one or two hairs. The fluid contained in them is not thick and glutinous, but, on the contrary, is rather thin and light coloured, drying with great rapidity; in these respects it differs very materially from the discharge of impetigo sparsa, which is thick and viscid, solidifying very slowly, and having all the characters of laudable pus. By its desiccation it gives rise to irregularly shaped, hard scabs, each of which is traversed by a hair: these, which are at first small, give a rough and rugged feel to the part of the scalp affected, but they quickly increase in size by the additions they receive from below. When once formed, they soon become detached, and are lifted off the scalp by the growth of the hairs which pass through them, and to which they are firmly adherent, appearing as it were strung upon them; they then dry, and sometimes become excessively hard, like grains of sand. The same process taking place again and again, each hair will be found to have several small scabs fixed upon it, at intervals of a line or two from one another. The colour of these scabs, which is a dirty greyish-white, passing into yellowish-gray and grayish-brown, has caused them to be compared, not inaptly, to pieces of old mortar, or of dirtied plaster; some of them also resemble, very closely, dried crumbs of bread. They are very irregular in their shape, but usually approach to a round or square figure.

The hair sometimes becomes light coloured, coarse, and woolly, on the affected parts ; as the bulbs however are not implicated, this change is not permanent."

The odour is exceedingly disgusting, and pediculi swarm to an amazing extent amongst and around the scabs.

*Impetigo Eczematosa* appears after much redness, tingling, and itching of the scalp. " A number of achorous pustules, of a yellowish-white colour, and closely set together, then make their appearance ; they are usually intermixed with a few vesicles, the contents of which however soon become opalescent and milky, and, in the course of twenty-four hours, purulent; but some of them occasionally remain unchanged until they are either ruptured by the patient scratching himself, or give way naturally. The eruption may occur in any part of the scalp, but its seat of election appears most frequently to be about the fore part of the head and temples.

The fluid that is effused on the rupture of these pustules is thick, viscid, and of a yellow or yellowish-green colour, very tenacious, glueing the hair together into masses, which have a soft, moist appearance, as they do not dry perfectly. When the pustules have once given way, small deep depressions are left, from which a viscid, semitransparent secretion continues to ooze, without, however, the evolution of any fresh eruption ; sometimes when this is very abundant it appears amongst the hairs in the form of drops, somewhat resembling impure honey.

Scabs are first formed on those parts of the head that are not covered by hair, as the forehead and temples, whence they may gradually extend themselves over the whole of the scalp or a great part of the face. These which are, in their earlier stages, thin and somewhat lamellar, soon acquire, from the constant additions they receive from the discharge that drains from a number of points, a thick and rugged appearance, and being of a yellow or yellowish-brown colour, semitransparent and shining on the surface, they resemble, sufficiently closely, dried honey, or pieces of yellow wax." Pediculi are apt to form. The hair is not permanently lost. Pruritus is greater in this than in any other affection of the scalp.

Mr. Erichsen gives an account of impetigo eczematosa of the face, which we do not think it necessary to notice. Age, he remarks, influences impetigo considerably. Impetigo eczematosa is more generally met with before the second or third year than at any subsequent period, whilst impetigo granulata and impetigo sparsa almost always occur after that age, namely, between the fourth and the seventh or eighth years, but rarely later than this. Temperament and habit of body, too, exert a great effect. Impetigo eczematosa and impetigo sparsa are most commonly met with in children of a lymphatic, lymphatico-sanguine, or sanguine temperament, who are frequently remarkable for their beauty, and otherwise healthy appearance, having thin, delicate, fair skins, light hair, blue or gray eyes, and a ruddy complexion ; whilst impetigo granulata, on the contrary, is most frequently seen in subjects of a melancholic or melancholico-nervous temperament, with dark complexion, hair, and eyes, and of a spare conformation.

Impetigo is more rife during the warm season of the year than at any other period, occasionally getting well during the Winter months to break out afresh on the return of Spring or Summer; but when chronic, season loses its influence.

Over-feeding, as well as under-feeding, and improper feeding severally lead to the disease. So do the exanthemata.

The duration of impetigo may vary from weeks to months, and even years, impetigo granulata being by far its most rebellious form. When it has once occurred it is very liable to relapses from trivial causes. No cicatrices or permanent baldness are ever left by it.

Our author makes some observations on the treatment. If mild, and in a young child, nothing, perhaps beyond simple palliatives, will be adviseable.

In a severer case, and in an older child, the first thing to be done is to remove the hair, which, as in eczema, may be accomplished more easily and with less irritation by means of a pair of curved blunt-pointed scissors, than with a razor. Then come fomentations and poultices, perhaps leeches, lancing of the gums, laxatives and so forth. The great thing is to avoid irritating.

When the case is chronic, the scalp must be cleared thoroughly of scabs, in order that more stimulating applications may be resorted to. Sulphureous lotions are of essential service. The sulphuret of potassium, in the proportion of from one to three drachms to a pint of water, applied four or five times a day, will be found most useful in giving a more healthy tone to the diseased cutis.

At the same time that this lotion is applied externally, the sulphureous waters, either natural, as those of Harrowgate, or artificial, may be administered internally with good effect. Lotions of alum, of the sulphates of copper, or of zinc, of the nitrate of silver, or of the bichloride of mercury, have been employed, but they are inferior to that of the sulphuret of potassium. Sometimes the ung. hyd. nitratis is of service. For violent itching Mr. Erichsen advises a lotion consisting of the oxide of zinc, suspended in rose water, to which a few drops of hydrocyanic acid have been added. Mild purgatives must not be forgotten.

"The diluted nitric or nitromuriatic acids in doses of from two drachms to half an ounce in the course of the day, will be found of much service in the treatment of this disease, being more especially of use in allaying the pruritus. They may be given in barley water or gruel, or in the form of a sherbet, with sugar or some agreeable syrup, as that of orange-peel." 103.

This has not agreed with our experience at all, the mineral acids generally aggravating the pruritus excessively. Nor does the statement that follows tally any better with what we have seen.

"I have never met with any cases of impetigo of the scalp that did not yield to the judicious employment of the above plan of treatment, or that necessitated the use of arsenical preparations, or of those remedies that are usually looked upon as *specifics* in the treatment of the diseases of the skin." 103.

We venture to say that the majority of medical men *will* meet with cases that *will not* yield to the above plan of management, which we consider rather meagre.

Circumscribed abscesses in the subcutaneous cellular tissue, and even deeper mischief may result from favus. The hair always becomes thin, dry, brittle, and frequently changed in colour, assuming a lighter hue than natural; but when the disease has not existed sufficiently long to destroy the bulbs, it will acquire its natural appearance and strength on a cure being effected; in some instances, however, the colour will be entirely lost, the hair becoming grey, and remaining so during the remainder of life. When the disease has gone on to the destruction of the bulbs, the hairs are of course never reproduced; the scalp therefore remains smooth, white, shining, and dry, and the part that is thus deprived of hair, will for ever afterwards be free from the disease itself, as with the destruction of the follicle, the seat of the affection, the disease itself must necessarily cease.

Favus dispersus has been met with on the face, nape of the neck, shoulders, extremities, and hands, nay, over the entire body, except the armpits and pubes, which, however general the disease may be, never become affected by it. On the scalp it is most obstinate.

*Favus Confertus.*

Favus confertus appears in the form of red circular patches, studded with a number of small yellow tubercles, which are, as in favus dispersus, depressed in the centre, and implanted as it were in the cutis. These, which are more numerous about the circumference of the patch than in its centre, are soon succeeded by crusts, which, although at first separate, and presenting the characteristic cupped shape, and circular outline, soon become confluent, and lose, to a certain degree, these characters, although they always retain a more or less round or oval form. They are always dry and friable, and at first of a yellowish colour, but this soon becomes grayish-yellow, and at last a dirty grayish-white, causing them to resemble, as Rayer justly observes, the crumbling mortar of a wall that is going to decay from age and moisture. When these scabs fall off, or are separated by poulticing, the skin under them will be seen to be red and shining, and fresh tubercles being speedily developed, a new succession of crusts takes place.

Favus confertus may spread in two ways. Either by the patches gradually extending their circumference by fresh evolutions of tubercles, which are succeeded by scabs, or else by new patches being formed, either by the patient, in scratching himself, inoculating other follicles with the favous matter, or else by the debris of the old scabs falling upon new places, and thus giving rise to a fresh eruption of the disease.

The hair is extensively affected, and, if baldness occurs, it is apt to be permanent. The clusters of this disease, increasing in size and number, soon unite by their margins, thus giving rise to large irregular patches, which sometimes occupy the whole of the vertex and fore-part of the scalp, usually leaving, however, as Willan observes, a border of hair round the head, unimpaired. When it has acquired this extent, the patient presents a remarkable appearance, the head being covered with a thick coating, as it were, of a dirty yellowish-gray or whitish plaster, which has entirely lost the cupped shape of the individual crusts, only retaining round its borders remains of its circular outline, in the form of segments of circles, of a greater or less extent, which are usually well marked, and have a bold, sweeping appearance. When the scabs fall off, the subjacent cutis will, at some points, be seen to be red, shining, and inflamed, whilst at others there will be bald, naked patches left, the hairs having been destroyed never to be reproduced.

In severe and chronic cases of the disease, the crusts will appear to be of a dirty white or grayish colour, friable, breaking readily into powder, with cracks and fissures in different directions, through which a thin, ichorous, fetid pus is exhaled, which is, however, perfectly distinct from favous matter, and is merely the produce of the inflamed skin. What little hair may still remain on the head will be thin, light-coloured, and woolly.

The patients, in severe cases, generally present all the marks of cachexia.

In spite of what Alibert may say, there can be no doubt of the contagiousness, and that extreme, of Favus. And its hereditary character would seem to be decided. Poverty, filth, the scrofulous diathesis, predispose to it.

*Treatment.*—This must consist of local and of general measures.

The *local treatment* presents three indications, viz.:

1st. To clear the scalp of all scabs and crusts, and to attend scrupulously to cleanliness.

2d. To remove the hair from the diseased follicle.

3d. To set up a new action in the part affected.

The first indication is readily fulfilled by the application of poultices continued for two or three days, or by means of lotions containing the diluted hydrochloric acid in the proportion of an ounce to the pint of water, after the hair has been cut short. Either of these means will suffice for the object in view, but the poultices are preferable, as they are always most convenient in their application and most speedy in their action. If, however, the crusts be very thick, they may first of all be loosened by the acid lotion, which acts by dissolving out their earthy parts, and then be separated by means of a large, thick, and soft bread or linseed-meal poultice, which will effectually clear the scalp. In the course of the treatment, cleanliness must be attended to by washing the head at least once a day with soap and water.

Unless the second indication is fulfilled, and the hairs are removed, no good can be effected. The old pitchcap is, happily, burnt, and even Plumbe's forceps have gone to rust. The depilatory of the Messrs. Mahon has superseded them, and although the composition is kept secret, and the remedy, therefore, a quack one, still it must be owned to be successful. Perhaps many of our readers are not acquainted with it. We shall therefore give our author's account of it. "They begin their treatment by cutting the hair at a distance of two inches from the scalp; the scabs are then removed by means of emollient applications and of poultices, and the skin freed from all impurities by means of soap and water. After this has been repeated for several days in succession, an ointment composed of lard and a depilatory powder, the composition of which is kept secret, is rubbed in every second day on the parts that are affected. A fine comb is then passed through the hair on the days on which this preparation is not used, and thus the hair is got rid of gradually and slowly, but without pain. After this plan has been continued for a few weeks, a small quantity of the powder is scattered through the hair, and the combing proceeded with. This is persevered in, according to the severity of the disease, for a longer or a shorter period, and has been found to succeed when every other mode of treatment has failed. It causes no pain, is devoid of danger, and does not prevent the hair from growing, provided the bulbs have not been destroyed. The composition of the ointment and powder is kept a secret, but according to Chevalier, who has analysed them, they consist of slaked lime partly carbonated, of a little silica, alumina, and oxide of iron (probably impurities in the lime), and of subcarbonate of potass; their activity evidently depending upon the lime and subcarbonate of potass they contain.

"For the ointment* of Mahon we may subsitute one composed of ʒj of carbonate of potass to ℥j of lard, or else a lotion containing ʒij to ʒiij of the same salt to ℥vj of water, either of which, if used in the way recommended by Mahon, will be found to act as mild and sure depilatories." 155.

Countless, almost, are the means recommended for carrying out the third indication, and setting up a new action in the scalp. Of all the applications, the iodide of sulphur, the sulphuret of potassium, and the carbonate of potass are incomparably the best. The former should be employed in the proportion of ten grains, or a scruple, to the ounce of lard. If the ointment be of a greater strength than this, it will be very apt to give rise to an eczematous affection of the scalp, by irritating it too powerfully; it may be used twice a day. Active as this preparation undoubtedly is, it occasionally fails, especially in chronic cases of the disease occurring in a scrofulous subject. We confess that we have seen no local application comparable to this.

"The sulphuret of potassium, in the form of lotion, is especially of service in removing that scurfy condition of the scalp that is left after the removal of the scabs.

Lotions of the carbonate of potass are also most useful, as they possess the double advantage of removing the hairs from the diseased follicles, at the same time that they excite the vessels of the scalp to a new and a more healthful action.

When favus occurs on the extremities or trunk, in the form of a few small tubercles only, it may most effectually be got rid of by cauterization with the nitrate of silver, or the strong mineral acids." 157.

* "M. Petel recommends the subjoined ointment and powder:

| | |
|---|---|
| Soda (of commerce) . . . | 60 parts |
| Slaked lime . . . . . . | 4 |
| Lard . . . . . . . | 120 |
| Mix together for the ointment. | |
| Quicklime . . . . . . | 120 parts |
| Powdered charcoal . . . | 8 |
| Mix for the powder." | |

We are glad to perceive that Mr. Erichsen advises the gentlest means. We cordially concur with him.

*General Treatment.*—On this Mr. Erichsen says little. When the disease arises spontaneously in a cachectic habit, *that* must be treated as the tubercular cachexia. Mr. E. lauds highly the iodide of iron. It may most conveniently be given to children in half-grain or grain doses, twice a day, in a tea-spoonful of simple syrup, which, at the same time that it conceals its taste, prevents its decomposition. Next to the iodide the ammonio-tartrate of iron, in doses of from two to five grains, will be found of most service, especially if given in combination with infusion of calumba, or the compound decoction of aloes, which, although not a very chemical mixture, is a very useful one. These preparations should, however, only be employed when the disease is ingrafted on a weak, scrofulous constitution, and are not called for when it occurs from contagion in a healthy subject. If any excitement supervene during their use, if the tongue should become dry and glazed, or red at the tip and margin, and the skin hot, they must immediately be discontinued, and a few doses of mercury and chalk, or of rhubarb and soda administered, so as to lessen the irritation.

And Mr. Erichsen concludes that, by attention to cleanliness and pursuing the plan of treatment recommended, there are few cases of favus that will not speedily be cured.

### Squamous Diseases.

Psoriasis and lepra *may* affect the head, but rarely unless they are present on other parts of the body. *Pityriasis* is more strictly a scalp disease. We perceive nothing in Mr. Erichsen's account of this affection to detain us. The following is his method of treatment. In children, he says, slightly stimulating washes, spirit or zinc lotions, with the daily use of a small-toothed comb, and a soft brush, will in general suffice; but when the disease is more chronic, especially when occurring in advanced life, we must have recourse to more active measures. In these cases, lotions of the sulphuret of potassium will be found exceedingly useful, more particularly if conjoined with the use of the ointment of the white precipitate of calamine, or of the oxide of zinc; the Harrowgate, or other sulphureous waters being at the same time taken internally, and due attention paid to the state of the secretions, especially those of the liver and alimentary canal.

The head should be washed daily with soap and water, and the scales removed with a soft brush, the hair being at the same time cut very short. Mild measures of this description, carried on with patience and perseverance, will seldom fail in effecting a cure, which is in many cases retarded by the employment of irritating ointments and stimulating lotions.

### *Alopecia.*

Alopecia may either be a primary affection of the hair-bulbs, being unconnected with any other lesion, or it may be secondary, being the sequela of some other disease, whether vesicular, pustular, or tubercular, of the scalp. It may be general or partial, slow or rapid in its progress. It is most frequent in the old, an effect of age; whilst, in early life, it is a consequence of some abnormal condition of the hair-bulbs. It is much more common in men than women.

Primary alopecia is of three kinds:

1st. That dependent upon atony or atrophy of the hair-bulbs.

2d. *Alopecia folliculosa,* depending upon a morbid condition of the hair-follicles. The *teigne tondante* of Mahon and Rayer.

3d. *Alopecia circumscripta,*—the porrigo decalvans of Willan.

"1. Alopecia, depending upon atony or atrophy of the secreting organs of the hairs, may arise from various causes. Old age, any circumstance that debilitates the system generally, as long-protracted illness, phthisis, dyspepsia, profuse discharges, venereal excesses, over-study, the depressing passions, fevers of a low type, and in fact anything that lowers the vital energies, thus influencing more especially the activity of the extreme parts of the body, may occasion it. Long-continued pressure, as from a military cap or helmet, may also give rise to it. In some rare instances there appears to have been a congenital atony of the hair-bulbs, not only of the scalp but of the body generally; the surface being entirely destitute of hair, and only furnished with a light down. Rayer and Cullerier both cite instances of this description.

Syphilis, and the abuse of mercurials, have been said by some to give rise to alopecia, but this is probably erroneous, and is founded on popular opinion, the cases related being by no means unequivocal, but appearing rather to be the accidental complication of this disease with syphilis, than a consequence of that affection." 184.

We think that they who have seen much of syphilis will not doubt its giving rise to alopecia. Partial loss of the hair is very common in connexion with secondary symptoms, and sometimes the alopecia is very extensive indeed. We have seen the head, face, eyebrows, pubes stripped of hair, or only covered with a sort of down. And this is too frequent an occurrence to admit of its being looked on as merely a coincidence.

In these cases there is more or less of an atonic or atrophied condition of the hair-bulbs.

### *Alopecia Folliculosa.*

This rare affection "is characterized," says our author, "by the presence of circular or oval patches of variable extent, somewhat elevated above the surface of the surrounding scalp, and of a greyish, pink, or dull purple colour; thickly studded with a number of small papillæ, that give a rather rough feel to the part affected, the hairs covering which, are always broken off at a distance of two or three lines from the surface of the skin.

This disease makes its appearance, in the first instance, in the form of a small, greyish, scurfy patch on the scalp, a line or two in diameter, but which may enlarge to the size of the palm of the hand, or even extend over the greater part of the head. On a cursory inspection, the patch appears to be bald, but when examined more closely it will be found to be covered with hairs two or three lines in length. It is always of a pretty regularly circular or oval figure, and in some instances appears as if made up of two or more circles joined together. Its colour is uniformly a grayish-pink, or dull purple, and it is somewhat elevated above the surrounding skin. Its surface, which is rough, dry, and rather hard, is evidently deprived of those oily or sebaceous secretions that serve to soften the scalp when in a healthy condition, and is covered by a number of papillæ, which, being closely set, give it very much the appearance of a portion of the integument of the leg of the fowl. In consequence of the close arrangement of these papillar bodies the skin has a very dense and compact appearance, and when it is raised up between the fingers it feels as if hypertrophied, being both thicker and firmer to the touch than natural. The surface of the diseased patch is covered by a number of very thin, fine, small scales, of a light-brownish or silver-gray colour. These, from their minute size and light colour, occasionally give it a mealy appearance. This is more particularly the case with the smaller patches.

The causes of this affection are very obscure; it occurs chiefly in children, and appears sometimes to be contagious, but of this we have no direct evidence."

Mr. Erichsen agrees with Mahon in placing the disease in the sebaceous follicles of the scalp. He thinks it probable that the sebacious matter is accumulated in the orifice of the follicle, where it undergoes some change, becoming harder and firmer by the absorption of its more oily parts, and that it is this collection in the follicle that gives rise to a small, prominent tumor; a number of which being situated close together, will cause the skin to assume the close papillar arrangement that is so characteristic of the disease. That it is seated in the sebaceous follicle is rendered still more probable, by the remarkable dryness that the affected part of the scalp presents, which can only be accounted for by the absence of the oily secretion that it is the office of these organs to pour out; and it is probably owing to the same cause that the hairs become fragile and break off.

On *Alopecia Circumscripta,* the Porrigo Decalvans of Willan, our author communicates nothing new, save that according to Cazenave, an epidemic alopecia of this description made its appearance in most of the schools in Paris in the year 1839. It was not preceded by any pustule or vesicle, and occurred in different parts of the town much about the same time; there was no evidence of its being of a contagious nature.

With regard to *treatment* Mr. Erichsen observes that when " loss of hair depends upon mere atony of these organs, there being no inflammation or ulceration of them, as when it is the effect of low fevers, chronic diseases, such as dyspepsia and other similar complaints, stimulating washes or ointments may be of service, as soon as the morbid state of the system is rectified; for until this be accomplished, either no good effect will follow their application, or it will only be temporary. For this purpose stimulating lotions and embrocations, containing the essential oils of rosemary, thyme, lavender, mace, or turpentine, infusions of walnut-leaves and of mustard, and alcoholic washes, have been recommended. Willis has seen the mercurial ointment of service in some cases. Dr. Thomson recommends a lotion composed of alcohol and spirits of turpentine, and Copland an ointment of the balsam of Peru and oil of lavender. Others have advised the application of solutions of the sulphates of copper or of zinc in alcohol, or of the nitrate of silver, of the tinctures of capsicum and of cantharides ; in fact, anything that stimulates the scalp may be of service. I have occasionally seen the infusion of tobacco, as recommended by Zacutus Lusitanus, succeed when other means have failed; but I have found nothing more useful than the continual shaving of the head, until the hair assumes its natural strength and colour. At the same time that this is being done, any stimulating applications, more particularly those recommended by Drs. Thomson and Copland, may excite the piliferous bulbs to increased action. When alopecia is dependent upon, or connected with, any chronic disease, that may lower the energies of the system generally, we cannot hope for a permanent cure until this be remedied, and the health of the patient restored. These remarks apply to alopecia circumscripta as well as to that form of baldness that arises from simple atony of the hair-bulbs.

Alopecia folliculosa is a most rebellious affection, and one that will resist all the above plans of treatment. The chief object appears to be to stimulate the sebaceous follicles, so as to cause them to discharge the morbid secretion that is accumulated in them. For this purpose I employed, in the two cases already mentioned, the ointments of the iodide of sulphur, and of the nitrate of mercury, with some success. But yet I cannot speak confidently of any mode of treatment, nor can I find any recommended by others."

This concludes the work of which we have given a pretty full account. Its copiousness indeed is not so much on account of the absolute novelty of what the work contains, as because the descriptions of the diseases of the scalp are concise, accurate, and clear, and calculated to dispel an ignorance on their nature and treatment too prevalent in the profession.

We must confess that, as plain practical men, there seems to us too great an anxiety to distinguish varieties of the great forms of cutaneous disease. Look for example, at the named forms of herpes. Their treatment is very similar, and the great difficulty, in most instances, is to tell what the *name* of the disease is. If it were a mere matter of perplexity it would not signify. But whilst we are running after the shadow, we are like to lose the substance ; and when the object of our studies is to invent or to apply a designation to a disorder, we may not improbably fail to attain satisfactory and comprehensive views of its treatment. We think then that the great desideratum in describing cutaneous disease, is to simplify and reduce to general formulæ, rather than to extend and individualise. To take again the case of herpes. We believe that he will treat it best, who is acquainted with the leading characters of the eruption, and the state of system that it is connected with ; while it is very possible to christen every variety with scrupulous accuracy, and, attempting to suit a different treatment to each name, to miss the general principle that should govern all.

